# Plasma bioactive adrenomedullin predicts mortality and need for dialysis in critical COVID-19

**DOI:** 10.1038/s41598-024-74380-x

**Published:** 2024-10-11

**Authors:** Patrik Johnsson, Theodor Sievert, Ingrid Didriksson, Hans Friberg, Attila Frigyesi

**Affiliations:** 1 Department of Clinical Medicine, Anaesthesiology and Intensive Care, Lund University, 22185 Lund, Sweden; 2 Department of Intensive and Perioperative Care in Malmö, Skåne University Hospital, 20502 Malmö, Sweden; 3 Department of Intensive and Perioperative Care in Lund, Skåne University Hospital, 22185 Lund, Sweden

**Keywords:** Adult respiratory distress syndrome, COVID-19, Critical care, Adrenomedullin, Mortality, Dialysis, Predictive markers, Prognostic markers, Viral infection, Respiratory distress syndrome

## Abstract

COVID-19 is a severe respiratory disease affecting millions worldwide, causing significant morbidity and mortality. Adrenomedullin (bio-ADM) is a vasoactive hormone regulating the endothelial barrier and has been associated with COVID-19 mortality and other adverse events. This prospective cohort pilot study included 119 consecutive patients with verified SARS-CoV-2 infection admitted to two intensive care units (ICUs) in Southern Sweden. Bio-ADM was retrospectively analysed from plasma on ICU admission, and days 2 and 7. Information on comorbidities, adverse events and mortality was collected. The primary outcome was 90-day mortality, and secondary outcomes were markers of disease severity. The association between bio-ADM and outcomes was analysed using survival analysis and logistic regression. Bio-ADM on admission, day 2, and day 7 only moderately predicted 90-day mortality in univariate and multivariate Cox regression. The relative change in bio-ADM between sample times predicted 90-day mortality better even when adjusting for the SAPS3 score, with an HR of 1.09 (95% CI 1.04–1.15) and a C-index of 0.82 (95% CI 0.72–0.92) for relative change between day 2 and day 7. Bio-ADM had a good prediction of the need for renal replacement therapy in multivariate Cox regression adjusting for creatinine, where day 2 bio-ADM had an HR of 3.18 (95% CI 1.21–8.36) and C-index of 0.91 (95% CI 0.87–0.96). Relative changes did not perform better, possibly due to a small sample size. Admission and day 2 bio-ADM was associated with early acute kidney injury (AKI). Bio-ADM on ICU admission, day 2 and day 7 predicted 90-day mortality and dialysis needs, highlighting bio-ADM’s importance in COVID-19 pathophysiology. Bio-ADM could be used to triage patients with a risk of adverse outcomes and as a potential target for clinical interventions.

## Introduction

### Critical COVID-19

Critical Coronavirus disease (COVID-19) requiring intensive care is characterized by acute respiratory distress syndrome (ARDS)^1^ and multiorgan dysfunction, posing a significant risk of severe morbidity and mortality^2,3^. Identifying patients likely to develop critical disease and respiratory failure can aid resource allocation and adequate therapeutic interventions. The search for predictive biomarkers and clinical features that can forecast the disease course may be particularly important in COVID-19 research. SARS-CoV-2 enters the host via the angiotensin-converting enzyme 2 (ACE2) receptor, widely expressed in the lungs, endothelial cells, kidneys, and heart^4,5^. A key factor of COVID-19 pathophysiology may be that SARS-CoV-2 causes endothelitis and vasculopathy through endothelial dysfunction, causing vascular leakage, thrombosis, and impaired vascular blood flow. These factors are a driving force in organ dysfunction and oedema^6–8^.

### Adrenomedullin

Bioactive adrenomedullin (bio-ADM) is a vasoactive hormone in vascular homeostasis, preventing oedema formation and organ damage^9^. Bio-ADM was first isolated from human pheochromocytoma cells by Kitamura et al.^10^. It is found in several organs throughout the body, including the heart and kidney, and particularly in the lung^11,12^. Bio-ADM is extensively expressed in endothelial cells, with a role of inducing vasodilation and decreasing vascular leakage by stabilizing the endothelial barrier^9,13–15^. Bio-ADM has several precursor molecules. Pre-pro-ADM is converted to pro-ADM, then converted and released into circulation as the biologically inactive mid-regional pro-adrenomedullin (MR-proADM). MR-proADM is then converted to the biologically active hormone (bio-ADM) through a prohormone convertase and amidation of its C-terminal^16^. Since MR-proADM is only partially converted to bio-ADM to a highly variable degree, it does not accurately represent the amount of biologically active adrenomdullin^16^. Thus, bio-ADM should better represent the patient’s physiological status compared to MR-proADM and has been extensively studied in sepsis, where high levels of bio-ADM and its’ inactive precursor correlate with disease severity and mortality^17–19^. An intervention with bio-ADM or the antibody adrecizumab, which also increases bio-ADM in plasma, has been suggested as a future treatment of sepsis^20^.

### Adrenomedullin and critical COVID-19

Recently, several studies have shown a relationship between high levels of adrenomedullin and severity of illness in hospitalized COVID-19 patients^21–26^. Adrecizumab has been tested to treat severe COVID-19 ARDS in a small uncontrolled case series^27^. Studies of the association between adrenomedullin and COVID-19 have mainly been performed on MR-proADM^21–24^, and have consistently shown elevated levels in non-survivors compared to survivors^28^. In one study, MR-proADM levels on hospital admission showed excellent predictive value for 28-day mortality in COVID-19 patients with an AUC of 0.91 and were also highly predictive of progression to severe disease^21^. Other studies on MR-proADM have also shown good prediction with an area under the curve of approximately 0.80^22–24^, which suggests that bio-ADM has an important pathophysiological role in COVID-19.

Only two studies have investigated the association between bio-ADM and COVID-19 mortality. In a Dutch study of 80 patients, bio-ADM levels on day 7 from ICU admission predicted mortality with an AUC of 0.70 for 28-day mortality. In addition, Bio-ADM was predictive of acute kidney injury (AKI)^25^. In a small German study, similar results were shown for 28-day mortality with an AUC of 0.72, and there was also a strong association between bio-ADM, serum creatinine and the need for renal replacement therapy (RRT)^26^.

### Objectives

The primary aim of this study was to investigate the association between bio-ADM levels from serial samples and 90-day mortality in critical COVID-19. The secondary aim was to investigate the association between bio-ADM levels and disease severity, the need for and duration of mechanical ventilation, the need for RRT, acute kidney injury (AKI) and length of stay (LOS) in critical COVID-19.

## Methods

### Study design

This study was a pilot study. This prospective cohort is part of the larger SweCrit COVID-19 study^2^ (ClinicalTrials.gov: NCT04974-775). The SweCrit COVID-19 study was predetermined to end after one year (on May 10, 2021), and this study represents the first 119 patients. The methods below have previously been described and used in related studies^2,29^. Patients where COVID-19 was not the primary cause of ICU admission were excluded. Blood was sampled from the patients, and clinical data were collected from their medical records. If possible, written informed consent was obtained before ICU admission. If this was not feasible, samples were collected, and written informed consent was obtained after ICU discharge or up to one year later. If written informed consent could not be obtained, the patient was excluded, and the samples were destroyed.

All research was performed following relevant guidelines and regulations. The strengthening the reporting of observational studies in epidemiology (STROBE) guidelines were used^30^.

### Setting

This pilot study was part of a more extensive prospective multicentre cohort study of critically ill COVID-19 patients admitted to six intensive care units in the Skåne region of Sweden.

### Participants

All adult patients (18 years or older) with Polymerase chain reaction-confirmed SARS-CoV-2 infection admitted to any of the six participating ICUs in the Skåne region of Sweden between May 11, 2020, and November 30, 2020, were included when COVID-19 was the primary cause of admission.

### Variables

The primary outcome was mortality of all causes within 90 days of ICU admission. Secondary outcomes were ARDS severity measured by the ratio of partial pressure of oxygen in arterial blood to the fraction of inspiratory oxygen concentration (P/F ratio), duration of invasive mechanical ventilation, RRT, AKI, and ICU and hospital LOS.

Comorbidities were classified according to the updated Charlson Comorbidity Index^31^. The simplified acute physiology score 3 (SAPS-3)^32^ and the sequential organ failure assessment (SOFA) score^33^ were used for severity scoring.

ARDS was defined according to the Berlin definition^1^, with the modification that patients on high flow nasal oxygen (HFNO) treatment were also considered ARDS cases if they fulfilled the other criteria.

Acute kidney injury (AKI) was defined according to the Kidney Disease Improving Global Guidelines (KDIGO) Clinical Practice Guidelines for acute kidney injury^34^. The baseline creatinine was defined as the creatinine value before the start of intermediate or critical care. Only the first occurrence of AKI was included.

### Data sources/measurement

Background data and data regarding the ICU stay were collected from the regional COVID-19 quality register, which contains prospectively collected data from medical records. Missing data was retrospectively collected from medical records and the patient administrative system for intensive care units (PASIVA). Survival data was collected from the Swedish population register.

Blood samples were collected within six hours of ICU admission and on the morning of the second and seventh day of ICU stay. No further samples were obtained if the patient had been discharged from the ICU. Samples were excluded if they were taken later than six hours from ICU admission (for admission samples only) or if the freezing time was more than six hours. Blood was collected in EDTA vacutainers, centrifuged to obtain plasma, aliquoted, and frozen. The frozen plasma samples were stored in the SWECRIT biobank at Region Skåne. Samples were shipped, and batch analysis of bio-ADM was performed on thawed samples at the laboratory of SphingoTec GmbH (Hennigsdorf, Germany). This assay has previously been described^16^.

### Bias

Patients with missing bio-ADM samples were compared to those with complete samples. The interaction between the time from sampling to freezing and the bio-ADM value was analysed.

### Study size

As this was a pilot study, no power calculation was performed.

### Statistical analysis

For all hypothesis tests, we considered *p*-values <0.05 as significant. To assess a difference in the location of two independent variables, we used the Wilcoxon rank-sum test (Mann–Whitney U test). Differences in proportions were assessed using Pearson’s $\chi ^2$ test. Medians were reported with corresponding interquartile ranges (IQR), while the mean was reported with its standard deviation (SD). The number of missing observations was specified if a variable had missing values. The relationship between the two variables was examined using linear regression methods and the Spearman correlation coefficient. The natural logarithm was used if a parameter, due to skewness, needed transformation. When calculating the P/F ratio for a specific day, a trimmed mean was used, where 20% of the lowest and highest values were removed before calculating the mean. The association between bio-ADM and AKI was analysed using logistic regression and results are reported as odds ratios (OR) with 95% confidence intervals (CI). Cox proportional hazard regression was used for univariate and multivariate analysis of bio-ADM, survival, and RRT needs. The assumption of proportional hazards was tested. The results of Cox regression analyses are reported as hazard ratios (HR) with 95% confidence intervals (CI). The model predictive value was calculated using the model likelihood ratio chi-square statistic, and the concordance index (C-index) was used as an effect measure. Differences in Kaplan-Meier survival curves were assessed with the log-rank test.

## Results

### Participants

**Fig. 1:**
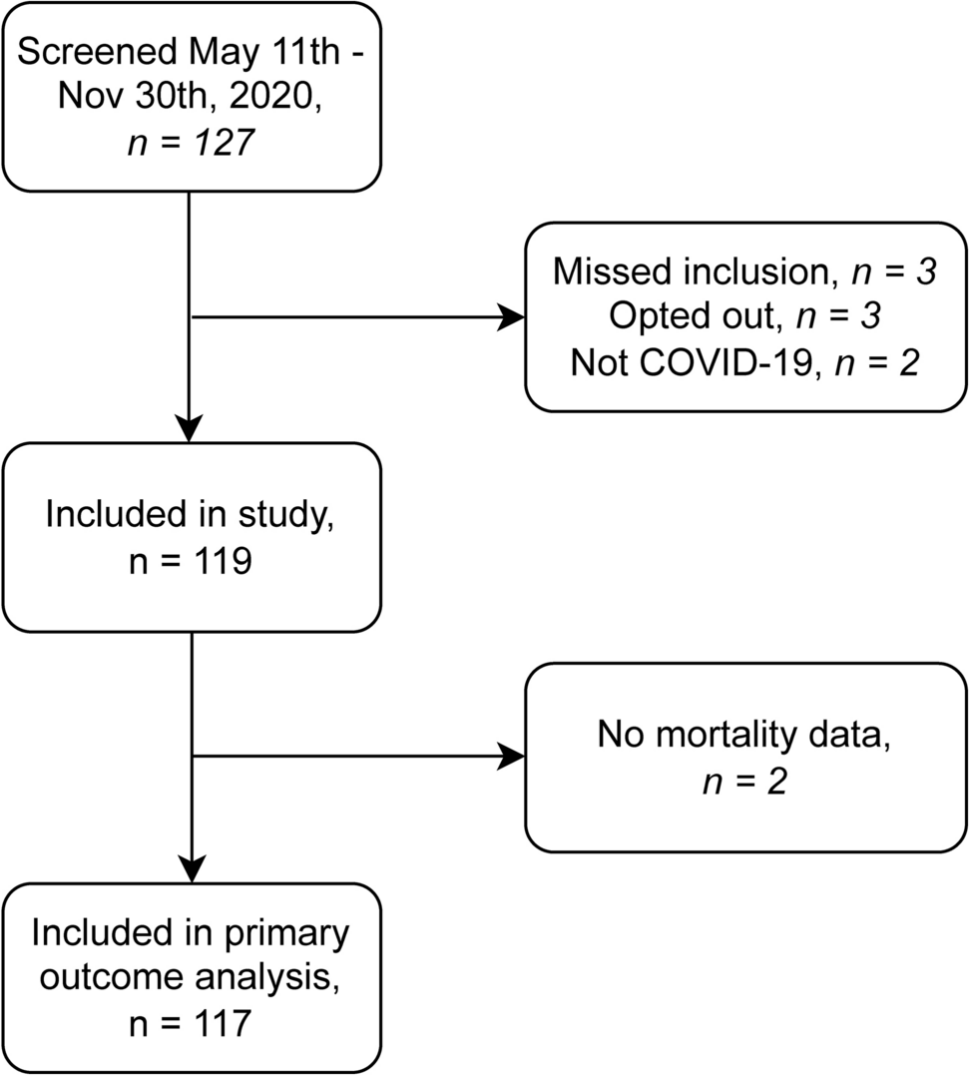
Study flowchart.

During the study period, 127 patients were screened for inclusion. Two patients tested positive for Severe acute respiratory syndrome coronavirus 2 (SARS-CoV-2) but were admitted for other reasons. Three patients declined participation, and three patients were initially excluded but later considered to fulfil inclusion criteria and thus characterised as missed inclusions. One hundred nineteen patients were included, and blood samples were taken; see Fig. 1. Of those included, Two patients (1.7 %) had missing mortality data in the population register and were excluded from the primary outcome analysis but had data regarding RRT.

### Demographics/descriptive data

Baseline characteristics are presented in Table 1. The overall 90-day mortality was 29% (34 out of 117). Non-survivors were older, with a median age of 71.0 years [66.3–80.5] vs. 62.0 years [52.5–68.0] in survivors (*p* < 0.001). Approximately 75% of the patients were male, with no difference between survivors and non-survivors. SAPS3-score was higher in non-survivors (62.5 [53.0–71.0] vs 51.0 [44.5–59.0], *p*<0.001). Non-survivors had a higher CCI, with a higher incidence of chronic obstructive pulmonary disease (COPD, 35.3% vs 15.7%, *p* = 0.035) and malignancy (17.6% vs 3.6%, *p* = 0.028). There was no difference in the proportion of active (3.6% vs 2.9%, *p* = 1.0) or previous (49.4% vs 61.8%, *p* = 0.3) smokers between survivors and non-survivors. Non-survivors were also slightly more frail, with a Clinical Frailty Scale (CFS) score of 3.5 [2.6–4.8] vs. 3.0 [2.0–3.0] (*p* = 0.002). Nearly all patients fulfilled the ARDS criteria. A total of 18 patients needed RRT in the ICU, with no difference between survivors and non-survivors. Among those who died, 25 (94.1%) died before, and 2 (5.9%) died after hospital discharge. In total, 22 out of 119 (18.4%) had or developed AKI on ICU admission or within the first day. The median time to AKI diagnosis was 1 day [0–1]. There was no difference in AKI incidence between survivors and non-survivors (19.3 % vs 17.6%, *p* = 1.0). Relative change in bio-ADM, expressed as mean (SD) per cent change, differed between survivors and non-survivors. Among survivors, bio-ADM decreased − 4.1% (11.8) from admission to day 2, − 9.1% (12.7) from admission to day 7, and − 6.9% (10.5) from day 2 to day 7. This contrasts with non-survivors, where bio-ADM increased 1.8% (11.0, *p* = 0.023) from admission to day 2, 1.12% (13.2, *p* = 0.004) from admission to day 7, and 3.2% (10.7, *p*<0.001) from day 2 to day 7. In patients who did not need RRT, mean (SD) change in bio-ADM was − 2.5% (11.36) from admission to day 2, − 8.1% (11.7) from admission to day 7, and − 5.5 (10.8) from day 2 to day 7. This differed from patients who needed RRT where mean (SD) change in bio-ADM was − 0.6% (14.3, *p* = 0.58) from admission to day 2, 1.9% (18.3, *p* = 0.014) from admission to day 7, and 3.5% (11.6, *p* = 0.015) from day 2 to day 7.

**Table 1:** Characteristics of survivors and non-survivors Data regarding general characteristics, illness severity, biomarkers and outcomes are presented below. Survivors were compared to non-survivors, and the *p*-values refer to that comparison. Proportions (%) are within their subgroups unless otherwise specified.

Parameter	Survivors	Non-survivors	*p* value
Admission characteristics			
n	83	34	
Age, years (median [IQR])	62.00 [52.50, 68.00]	71.00 [66.25, 80.50]	< 0.001
Sex = Male (%)	63 (75.9)	27 (79.4)	0.867
Body mass index, kg/m^2^ (median [IQR])	31.23 [26.91, 35.48]	28.55 [24.67, 31.63]	0.051
Temperature, $^{\circ }$C (median [IQR])	37.78 [37.30, 38.55]	37.35 [36.73, 38.00]	0.016
Duration of symptoms, days (median [IQR])	7.00 [5.00, 10.00]	6.00 [2.25, 10.00]	0.093
Admission SOFA score (median [IQR])	5.00 [3.70, 7.00]	6.65 [4.27, 8.00]	0.088
SAPS3 score (median [IQR])	51.00 [44.50, 59.00]	62.50 [53.00, 71.00]	< 0.001
Comorbidities			
Hypertension, n (%)	42 (50.6)	22 (64.7)	0.235
Smoker, active, n (%)	3 (3.6)	1 (2.9)	1.000
Smoker, previously, n (%)	41 (49.4)	21 (61.8)	0.311
COPD and severe asthma, n (%)	13 (15.7)	12 (35.3)	0.035
Charlson comorbidity index (median[IQR])	2.00 [1.0, 3.0]	4.50 [3.0, 6.0]	< 0.001
Clinical frailty scale (median[IQR])	3.0 [2.0, 3.0]	3.5 [2.6, 4.8]	0.002
Respiratory parameters			
ARDS, n (%)	80 (98.8)	31 (96.9)	1.00
pH Day 1 (min) (median [IQR])	7.43 [7.34, 7.46]	7.39 [7.31, 7.44]	0.060
PaO2 Day 1 (min), kPa (median [IQR])	7.40 [6.55, 7.90]	7.25 [6.25, 8.07]	0.957
PaCO2 Day 1 (max), kPa (median [IQR])	5.20 [4.80, 6.30]	5.51 [4.62, 6.38]	0.655
P/F-ratio Day 1 (min), kPa (median [IQR])	12.00 [10.00, 15.66]	13.00 [10.25, 16.97]	0.390
Biomarkers			
Bio-ADM_Admission_, pg/mL (median[IQR])	51.37 [31.92, 77.79]	69.89 [46.43, 102.16]	0.008
Bio-ADM_Day 2_, pg/mL (median[IQR])	45.87 [29.15, 66.48]	69.85 [44.28, 108.13]	0.006
Bio-ADM_Day 7_, pg/mL (median[IQR])	36.43 [23.80, 52.33]	57.94 [39.62, 91.72]	0.009
IL-6, ng/L (median [IQR])	105.47 [52.00, 206.39]	238.93 [84.25, 1151.45]	0.005
CRP, mg/L (median [IQR])	142.00 [93.00, 185.50]	156.50 [103.75, 180.50]	0.708
Creatinine, umol/L (median [IQR])	76.00 [66.00, 99.20]	95.00 [76.09, 142.50]	0.003
Leukocytes, × 10^9^/L (median [IQR])	8.70 [6.60, 10.83]	10.61 [8.22, 14.60]	0.036
Platelets, × 10^9^/L (median [IQR])	269.00 [197.50, 360.97]	205.50 [159.25, 264.56]	0.003
Treatment in the ICU			
Invasive mechanical ventilation, n (%)	40 (48.2)	22 (64.7)	0.155
Time to intubation, days (median [IQR])	1.67 [0.26, 2.28]	0.95 [0.53, 2.20]	0.871
Duration of IMV, days (median [IQR])	5.56 [4.39, 11.01]	9.23 [4.66, 16.38]	0.138
Prone position, n (%)	62 (74.7)	21 (61.8)	0.240
CRRT, n (%)	11 (13.3)	7 (20.6)	0.474
Time to CRRT, days (median [IQR])	11.69 [7.01, 12.78]	8.88 [6.70, 13.20]	0.922
LOS ICU, days (median[IQR])	6.9 [3.3, 14.0]	11.6 [2.3, 20.6]	0.460
Acute kidney injury, n (%)	16 (19.3)	6 (17.6)	1.00
Time to AKI, days (median[IQR])	0.5 [0.0–1.0]	1.0 [1–0–1.0]	0.15
ICU mortality, n (%)	N/A	23 (67.6)	< N/A
Hospital mortality, n (%)	N/A	32 (94.1)	< N/A

SD, standard deviation; IQR, interquartile range; CCI, Charlson Comorbidity Index; SAPS3, simplified Acute Physiology Score III; SOFA, sequential organ failure assessment; IMV, invasive mechanical ventilation; bio-ADM, circulating bioactive adrenomedullin; CRP, C-reactive protein; IL-6, interleukin 6; PCT, procalcitonin; LD, lactate dehydrogenase; IMV, invasive mechanical ventilation; RRT, renal replacement therapy; LOS, length of stay; AKI, Acute kidney injury; ICU, intensive care unit.

### Bio-ADM and mortality

**Fig. 2:**
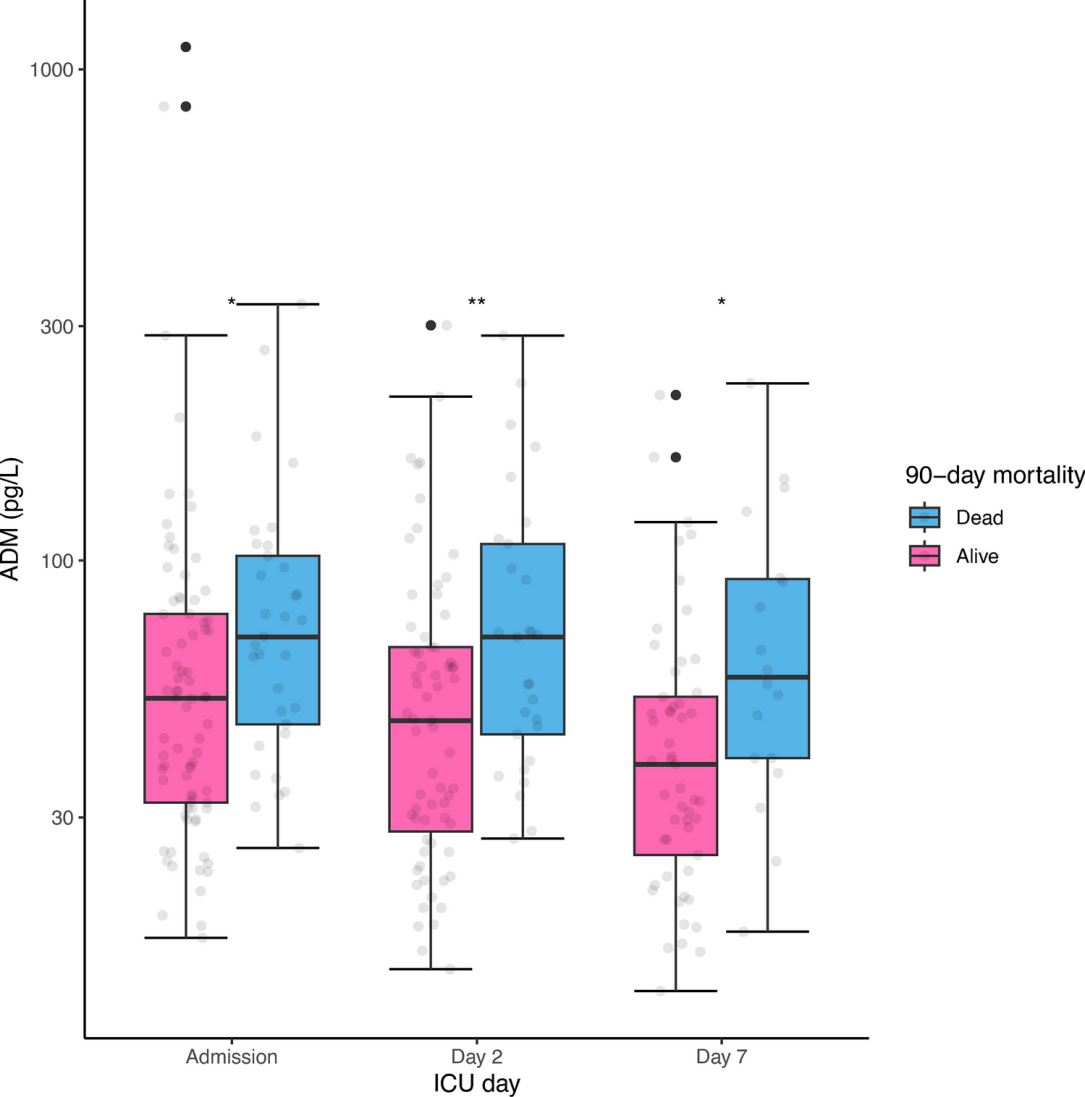
Bio-ADM in survivors and non-survivors of severe COVID-19. Bio-ADM: circulating bioactive adrenomedullin; ICU: intensive care unit; *: *p*-value < 0.05; **: *p*-value < 0.01.

Higher median bio-ADM values were seen in non-survivors both on admission (69.9 pg/L [46.4–102.2 pg/L] vs. 51.4 pg/L [31.9–77.8 pg/L], *p* = 0.008), day 2 (69.9 pg/mL [44.3–108.1 pg/L] vs. 45.9 pg/mL [29.1–66.5 pg/L], *p* = 0.006) and day 7 (57.9 pg/mL [39.6–91.7 pg/L] vs. 36.4 pg/mL [23.8–52.3 pg/L], *p* = 0.009). The fractions of missing bio-ADM values were 4.2%, 16.8%, and 38.7%, respectively. See Fig. 2.

**Fig. 3:**
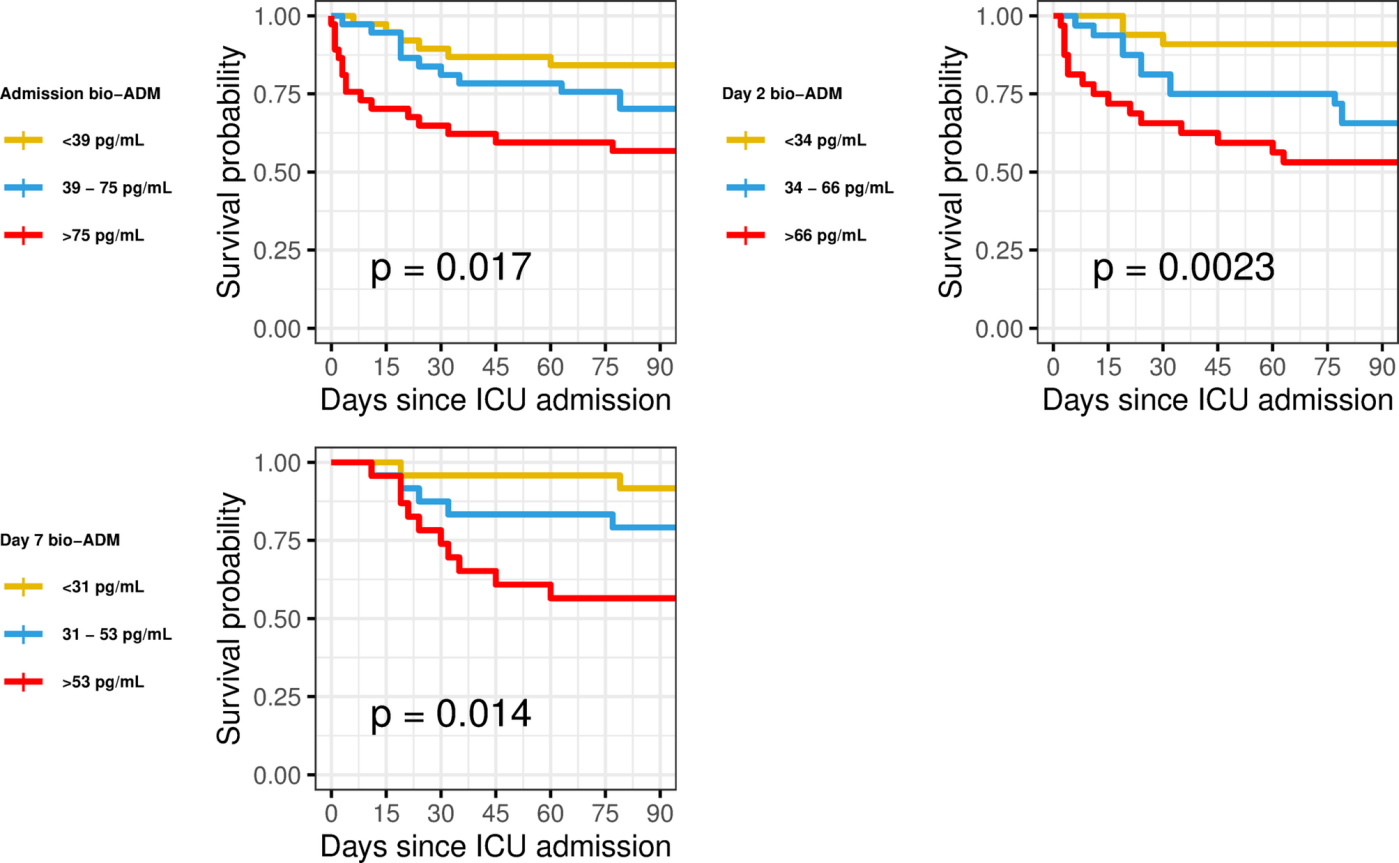
Bio-ADM tertiles and survival in severe COVID-19, from ICU admission, day 2, and day 7. Bio-ADM: circulating bioactive adrenomedullin; ICU: intensive care unit.

Bio-ADM was associated with 90-day mortality in univariate Cox regression analyses at all three sampling times. The admission day sample had the lowest hazard ratio (HR 1.54, 95% CI 1.1–2.2, *p* = 0.02) compared to day 2 (HR 2.2, 95% CI 1.3–3.6, *p* = 0.003) or day 7 (HR 2.5, 95% CI 1.3–4.8, *p* = 0.007). All three models were predictive of 90-day mortality with a C-index of 0.66 (95% CI 0.57–0.75), 0.67 (95% CI 0.57–0.77), and 0.69 (95% CI 0.56–0.81) for ICU admission, day 2, and day 7, respectively, see Table 2. In Kaplan-Meier plots, survival was lowest with the third bio-ADM tertile at all three sample times. See Fig. 3.

**Table 2:** Cox regression on bio-ADM and mortality. Univariate Cox regression using bio-ADM as exposure and 90-day mortality as outcome. Multivariate Cox regression using bio-ADM as exposure and adjusting for SAPS3. Hazard ratios are presented with their 95% CI, and the discriminative ability of the model is represented by the concordance statistic .

90-day mortality			
Parameter	Hazard ratio (95% CI)	*p* value	C-index (95% CI)
Univariate			
Admission bio-ADM	1.54 (1.06–2.24)	0.023	0.66 (0.57–0.75)
Day 2 bio-ADM	2.18 (1.31–3.65)	0.0029	0.67 (0.57–0.77)
Day 7 bio-ADM	2.48 (1.28–4.79)	0.0070	0.69 (0.56–0.81)
SAPS3	1.08 (1.05–1.11)	< 0.001	0.73 (0.65–0.82)
Bio-ADM, % change day 2 vs admission	1.04 (1.01–1.07)	0.020	0.63 (0.53–0.72)
Bio-ADM, % change day 7 vs admission	1.05 (1.01–1.08)	0.0049	0.70 (0.58–0.81)
Bio-ADM, % change day 7 vs day 2	1.10 (1.04–1.15)	< 0.001	0.74 (0.62–0.85)
Multivariate adjusting for SAPS3			
Admission bio-ADM	1.48 (0.91–2.43)	0.12	0.75 (0.67–0.83)
Day 2 bio-ADM	1.63 (0.93–2.86)	0.087	0.76 (0.67–0.84)
Day 7 bio-ADM	2.02 (0.95–4.26)	0.066	0.78 (0.67–0.88)
Bio-ADM, % change day 2 vs admission	1.03 (0.99–1.06)	0.011	0.79 (0.65–0.93)
Bio-ADM, % change day 7 vs admission	1.04 (1.01–1.07)	0.017	0.78 (0.68–0.88)
Bio-ADM, % change day 7 vs day 2	1.09 (1.04–1.15	< 0.001	0.82 (0.72–0.92)

CI, confidence interval; bio-ADM, circulating bioactive adrenomedullin; C-index, concordance statistic.

In multivariate Cox regression analyses adjusting for SAPS3, bio-ADM was not associated with mortality on admission (HR 1.48, 95% CI 0.91–2.43, *p* = 0.12), day 2 (HR 1.63, 95% CI 0.93–2.86, *p* = 0.087), or day 7 (HR 2.02, 95% CI 0.95–4.26, *p* = 0.066).

Using univariate Cox regression and looking at changes in ADM values over time, the relative increase in bio-ADM between day 2 and 7 was strongly associated with mortality (HR 1.10, 95% CI 1.04–1.15, *p* < 0.001), as was the relative increase from admission to day 7 (HR 1.05, 95% CI 1.01–1.08, *p* = 0.0049), and admission to day 2 (HR 1.04, 95% CI 1.01–1.07, *p* = 0.020). The C-index was 0.74 (95% CI 0.62–0.85) for relative change between day 2 and 7, 0.70 (95% CI 0.58–0.81) for relative change between admission and day 7, and 0.63 (95% CI 0.53–0.72) for relative change between admission and day 2.

The association between dynamic changes and mortality remained when adjusting for SAPS3 in multivariate Cox regression for change from day 2 to day 7 (HR 1.09, 95% CI 1.04–1.15, *p* < 0.001) and for change from admission to day 7 (HR 1.04, 95% CI 1.01–1.07, *p* = 0.017), but not for change from admission to day 2 (HR 1.03, 95% CI 0.99–1.06, *p* = 0.11). The models also predicted mortality with C-indices of 0.82 (95% CI 0.72–0.92) for change from day 2 to day 7 and 0.78 (95% CI 0.68–0.88) for change from admission to day 7, respectively.

### Bio-ADM and ARDS severity

Bio-ADM on admission, day 2, or day 7 was not associated with severe ARDS (P/F-ratio < 13) up to 7 days after sampling.

### Bio-ADM and RRT

In patients who needed RRT during their ICU stay, median bio-ADM was significantly higher on ICU admission, 88.2 pg/L [64.8–141.4 pg/mL] vs. 50.3 pg/mL [33.0–77.2 pg/L], *p* < 0.001), on day 2 (94.4 pg/L [71.5–160.4 pg/mL] vs. 46.4 pg/L [29.7–66.5 pg/mL], *p* < 0.001), and day 7 (119.7 pg/mL [67.3–146.7 pg/mL] vs. 35.7 pg/L [24.1–49.6 pg/mL], *p* < 0.001). Median bio-ADM increased over time (in consecutive samples) in the RRT group, while bio-ADM in the non-RRT group decreased over time.

**Fig. 4:**
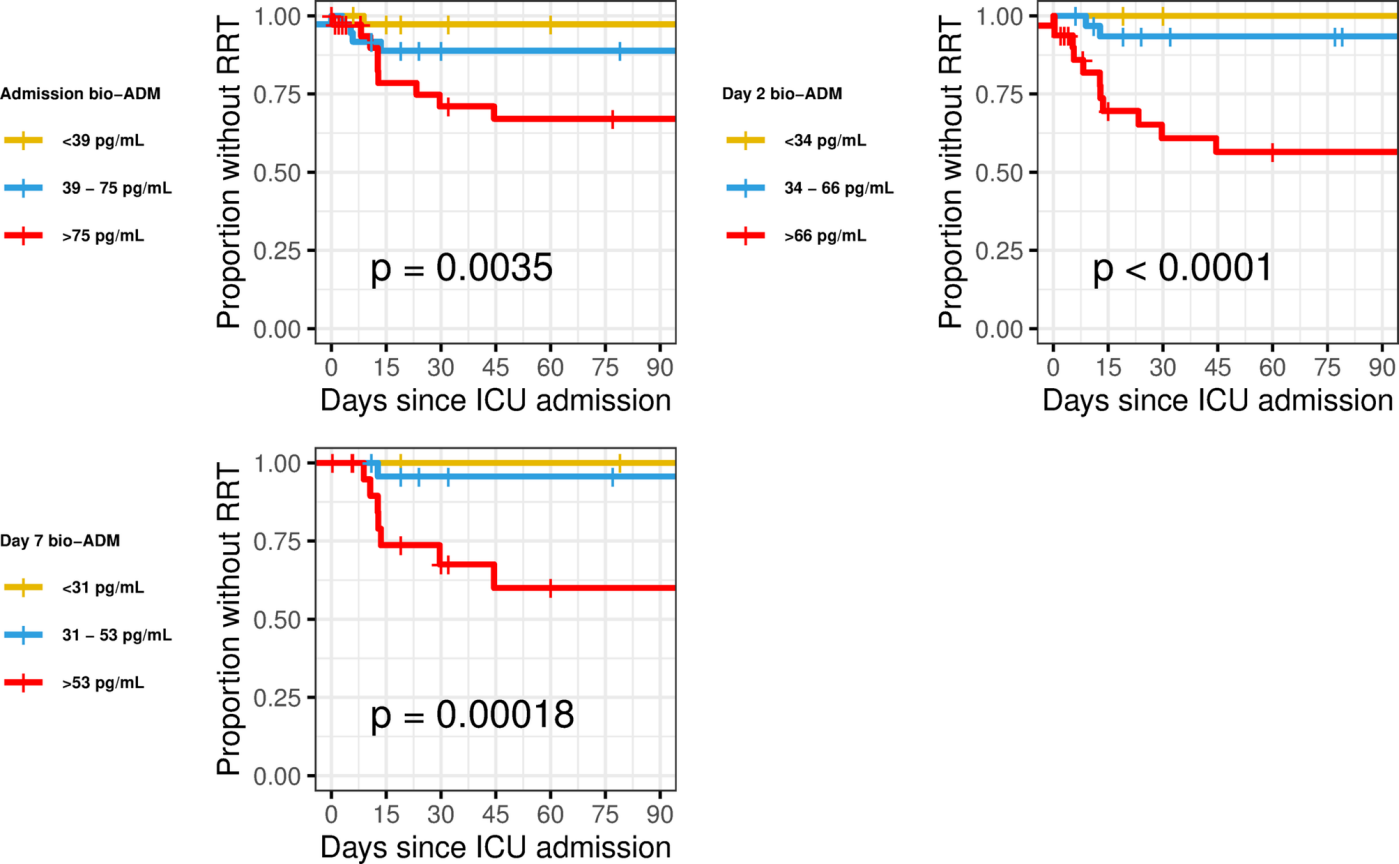
Bio-ADM tertiles and RRT-free survival, from ICU admission, day 2, and day 7. Bio-ADM: circulating bioactive adrenomedullin; RRT: renal replacement therapy; ICU: intensive care unit.

In univariate Cox regression analyses, bio-ADM on ICU admission (HR 2.78, 95% CI 1.75–4.41, *p* < 0.001), day 2 (HR 5.28, 95% CI 2.49–11.16, *p* < 0.001), and day 7 (HR 20.93 95% CI 4.95–88.63, *p* < 0.001) were associated with the need for RRT. Furthermore, all bio-ADM samples were predictive of the need for RRT with C-index of 0.77 (95%CI 0.66–0.88), 0.84 (95%CI 0.75–0.92), and 0.89 (95%CI 0.81–0.97) for admission, day 2, and day 7 samples, respectively, see Table 3. In Kaplan-Meier plots, the need for RRT increased with increasing bio-ADM tertile. See Fig. 4.

**Table 3:** Cox regression on bio-ADM and renal replacement therapy. Univariate Cox regression using bio-ADM as exposure and RRT. Multivariate Cox regression using bio-ADM as exposure and adjusting for creatinine. Hazard ratios are presented with their 95% CI, and the discriminative ability of the model is represented by the concordance statistic.

Renal replacement therapy			
Parameter	Hazard Ratio (95% CI)	*p* value	C-index (95% CI)
Univariate			
Admission bio-ADM	2.78 (1.75–4.41)	<0.001	0.77 (0.66–0.88)
Day 2 bio-ADM	5.28 (2.49–11.16)	<0.001	0.84 (0.75–0.92)
Day 7 bio-ADM	20.93(4.95–88.63)	<0.001	0.89 (0.81–0.97)
Admission creatinine	3.44 (2.03–5.82)	<0.001	0.71 (0.58–0.85)
Day 2 creatinine	4.94 (2.91–8.42)	<0.001	0.90 (0.85–0.94)
Day 7 creatinine	10.43 (4.06–26.82)	0.0028	0.91 (0.85–0.97)
Bio-ADM, % change day 2 vs admission	1.02 (0.98–1.07)	0.30	0.61 (0.43–0.80)
Bio-ADM, % change day 7 vs admission	1.07 (1.02–1.11)	0.0053	0.66 (0.46–0.86)
Bio-ADM, % change day 7 vs day 2	1.06 (0.99–1.14)	0.084	0.66 (0.48–0.84)
Multivariate adjusting for creatinine			
Admission bio-ADM	2.43 (1.26–4.69)	0.0082	0.81 (0.71–0.91)
Day 2 bio-ADM	3.18 (1.21–8.36)	0.019	0.91 (0.87–0.96)
Day 7 bio-ADM	6.54 (1.33–32.25)	0.021	0.94 (0.90–0.98)
Bio-ADM, % change day 2 vs admission	1.04 (1.00–1.09)	0.075	0.79 (0.65–0.93)
Bio-ADM, % change day 7 vs admission	1.08 (1.03–1.13)	0.0011	0.89 (0.78–0.99)
Bio-ADM, % change day 7 vs day 2	1.03 (0.98–1.09)	0.27	0.95 (0.90–1.00)

CI, confidence interval; bio-ADM, circulating bioactive adrenomedullin; RRT, renal replacement therapy; C-index, concordance statistic.

In multivariate Cox-regression adjusting for creatinine sampled concurrently with bio-ADM, bio-ADM was still associated with the need for RRT; HR 2.43 on admission (95%CI 1.26–4.69, *p* = 0.0082), HR 3.18 at day 2 (95%CI 1.21–8.36, *p* = 0.019), and HR 6.54 at day 7 (95%CI 1.33–32.25, *p* = 0.021). The models were also predictive of RRT need with C-indices of 0.81 (95% CI 0.71–0.91), 0.91 (95% CI 0.87–0.96), and 0.94 (95%CI 0.90–0.98) for admission, day 2, and day 7, respectively.

In univariate Cox regressions of relative change in bio-ADM between samples and RRT, there was an association with the relative change in bio-ADM from admission to day 7 and RRT need (HR 1.08, 95%CI 1.03–1.13, *p* = 0.0011) with a C-index of 0.66 (0.46–0.86). There was no association with relative change from admission to day 2 (HR 1.02, 95% CI 0.98–1.07, *p* = 0.3) or with change from day 2 to day 7 (HR 1.06, 95% CI 0.99–1.14), *p* = 0.084). In multivariate Cox regression adjusting for creatinine sampled on the same day, the relative change in bio-ADM from admission to day 7 was associated with RRT (HR 1.08, 95% CI 1.03–1.13, *p* = 0.0011) with a C-index of 0.89 (95% CI 0.78–0.99). Relative change between day 2 and admission (HR 1.04, 95% CI 1.00–1.09, *p* = 0.075) and between day 7 and day 2 (HR 1.03, 95% CI 0.98–1.09, *p* = 0.2) were not associated with RRT when adjusting for creatinine.

### Bio-ADM and AKI

Using logistic regression analysing bio-ADM on admission and day 2 and the presence or development of AKI on ICU admission, both admission bio-ADM (Odds ratio [OR] 3.37, 95% CI 1.70–7.63, *p* < 0.001) and day 2 bio-ADM (OR 2.92, 95% CI 1.34–6.82, *p* = 0.009) were associated with AKI in univariate analysis. In multivariate logistic regression adjusting for SOFA score, both admission bio-ADM (OR 2.84, 95% CI 1.43–6.35, *p* = 0.005) and day 2 bio-ADM (OR 2.87, 95% CI 1.26–7.05, *p* = 0.01) were associated with AKI.

### Bio-ADM and IMV and hospital LOS

Patients who required invasive mechanical ventilation (IMV) had higher bio-ADM median values on day 2 (57.3 pg/mL [41.5–88.3 pg/mL] vs 34.9 [25.5–61.8 pg/mL], *p* = 0.003) and day 7 (49.4 pg/mL [31.5–85.5 pg/mL] vs 34.4 pg/mL [21.3–47.8 pg/mL], *p* = 0.004) than those who did not. The median bio-ADM value on ICU admission did not differ between those who needed IMV and those who didn’t (61.0 pg/mL [37.8–83.3 pg/mL] vs 45.5 pg/mL [31.5–84.6 pg/mL], *p* = 0.164). In linear regression analyses, there was no association between bio-ADM on ICU admission, day 2 or day 7, and the duration of mechanical ventilation. Likewise, there was no association between bio-ADM on ICU admission or day 2 and the hospital LOS in linear regression analyses. However, there was a weak association between bio-ADM on day 7 and hospital LOS (r^2^ = 0.06, *p* = 0.037).

### Bias

Patients with missing bio-ADM samples had similar demographics and comorbidities as patients without missing bio-ADM values. Patients with missing values had a shorter duration of IMV, shorter ICU LOS, and a shorter hospital LOS. Ninety-day mortality did not differ (25% vs 12.1%, *p* = 0.21). Out of 357 total samples, 71 (19.9%) were missing for various reasons; 15 (4.2%) were due to the patient being deceased, 11 (3.1%) were due to discharge from the ICU, 2 (0.6%) were due to transfer out of a participating ICU, 1 (0.3%) because the patient declined further sampling, 34 (9.5%) samples were missing or mishandled, and 8 (2.2%) samples were missing for unknown reasons. See Table 4. There was no correlation between the time from sampling to freezing and the bio-ADM value (correlation coefficient, R = 0.14, *p* = 0.068).

**Table 4:** Missing bio-ADM samples. Data on reasons for missing bio-ADM samples. Proportions (%) are related to the total number of samples (n = 357).

Missing bio-ADM samples				
Reason for missing	ICU admission, n(%)	Day 2, n(%)	Day 7, n(%)	Total
Deceased	0	4 (1.1)	11 (3.1)	15 (4.2)
Discharged	0	0	11 (3.1)	11 (3.1)
Transferred	0	0	2 (0.6)	2 (0.6)
Declined further participation	0	0	1 (0.3)	1 (0.3)
Sample missing or destroyed	4 (1.1)	13 (3.6)	17 (4.8)	34 (9.5)
Unknown	1 (0.3)	3 (0.8)	4 (1.1)	8 (2.2)
Sum	4 (1.1)	17 (4.8)	42 (11.8)	71 (19.9)

bio-ADM, circulating bioactive adrenomedullin; ICU, intensive care unit.

## Discussion

In this pilot study of 119 critically ill COVID-19 patients in the ICU, bio-ADM was associated with 90-day mortality. Non-survivors had higher bio-ADM values on ICU admission and days 2 and 7. In addition, bio-ADM also predicted the need for RRT and was associated with AKI. Dynamic changes in bio-ADM had a strong association with both outcomes, where relative increases in bio-ADM were associated with mortality and the need for RRT.

There was no association between bio-ADM values and disease severity up to one week after sampling. Though bio-ADM was higher in patients who needed IMV, it was not associated with the duration of mechanical ventilation at any sampling time. There was a weak association between hospital LOS and bio-ADM on day 7.

This pilot study has several limitations. Patients were from six ICUs in southern Sweden, and our results should be confirmed in a larger and more diverse cohort. This study uses a broad definition of ARDS by including patients on HFNO who are not traditionally defined as ARDS patients. This approach has been suggested in the recent ARDS guidelines from the European Society of Intensive Care Medicine (ESICM)^35^. This study, however, aimed to investigate mortality in critical COVID-19 and not in ARDS specifically. Even though bio-ADM on ICU admission and later on were associated with mortality, no association with severe ARDS was seen. Sampling at hospital admission would probably have been better for predicting progression to severe disease and mortality. Many patients admitted to the ICU already had severe ARDS (as shown by the mean P/F-ratio on day 1), which is why this study cannot answer whether bio-ADM can predict progression to severe disease. Likewise, even though bio-ADM on day 7 and hospital LOS were associated, this may be irrelevant since patients who had taken an ICU sample on day 7 had already spent considerable time in the hospital. A significant number of bio-ADM values were missing, in particular on day 7. Some were deceased, and others were discharged from the ICU, where samples could not be obtained due to the high workload. See the discussion below. Even though this is the largest study on bio-ADM and COVID-19 to date^25,26^, the sample size was still relatively small and should be repeated with a larger sample.

Bio-ADM levels were higher in non-survivors at all time points, and increasing bio-ADM levels were associated with increased mortality. Since high adrenomedullin levels are related to endothelitis of other causes^36^, high bio-ADM levels in non-survivors may represent a more severe form of endothelitis in critical COVID-19^6,7,37^. Bio-ADM had limited predictive value for 90-day mortality with a C-index of 0.66 to 0.69, increasing slightly with sampling time. This is broadly similar to other studies of bio-ADM and COVID-19 mortality^25,26^. It is not surprising that day 7 samples had a stronger association with mortality than ICU admission samples since these patients likely represent a population with more severe disease.

In this study and other studies of bio-ADM, bio-ADM was predictive of mortality in critical COVID-19 but did not perform as well as MR-pro-ADM in distinguishing survivors from non-survivors. This is surprising since there is a clear biological rationale for choosing bio-ADM due to the variable conversion rate of the pro-hormone MR-proADM. A potential explanation may be that MR-pro-ADM has a strong association with age and creatinine^38^, both of which are associated with mortality in COVID-19^2^. Of note, all studies on bio-ADM and COVID-19 (including this study) use ICU admission as timing of first sample^25,26^, while many of the studies involving MR-pro-ADM have had sampling on hospital admission^21–23^. Thus, part of the reason for the inferiority of bio-ADM may be the timing of the sample and the actual population studied (hospitalised versus critically ill COVID-19 patients), indicating that the results are not entirely comparable.

Bio-ADM values from all three sampling times showed good predictive value of initiating renal replacement therapy in univariate analysis, with C-indices ranging from 0.77 to 0.89. Patients who later needed dialysis tended to have higher bio-ADM values over time (in consecutive samples). Conversely, patients who did not need RRT had bio-ADM values that decreased with time. These associations were still present when adjusting for creatinine taken on the same day as the bio-ADM sample. Other studies of bio-ADM and COVID-19 have also demonstrated an association between bio-ADM, admission creatinine, and AKI^25^, and the need for renal replacement therapy^26^. A study on MR-proADM and the need for dialysis in COVID-19 patients in the ICU also showed an association^39^.

Many patients had manifested AKI or developed AKI soon after ICU admission. In logistic regression adjusting for SOFA-score, there was a strong association between bio-ADM taken on admission or day 2 and AKI. We did not investigate the relationship between bio-ADM on day 7 and AKI since all patients who developed AKI did so before day 7. A more detailed study of bio-ADM and AKI status over time is beyond the scope of this study. The relationship between high bio-ADM values and kidney injury may involve COVID-19-caused endothelitis, and it is also hypothesised that AKI in COVID-19 is due to a combination of inflammatory responses and dysregulation of the renin-angiotensin system^40^. However, the exact pathophysiology of AKI in COVID-19 is still unknown.

Interestingly, dynamic changes in bio-ADM over time were associated with both primary and secondary outcomes. Relative bio-ADM change was associated with, and predictive of, 90-day mortality when adjusting for SAPS3, indicating that bio-ADM has a role in COVID-19 mortality. Models using relative changes performed better than models using absolute bio-ADM values in predicting 90-day mortality for critically ill COVID-19 patients. Dynamic changes did not predict RRT need well, possibly due to the relatively few events in our sample. However, the relative difference between admission and day 7 bio-ADM was strongly associated with RRT, even when adjusting for creatinine. These results highlight the importance of serial sampling, where rising values over time seem to predict a worse outcome. Ideally, increasing bio-ADM should enable clinical interventions in the future.

Bio-ADM was neither associated with severe ARDS in this study nor with a longer ICU stay, hospital stay, or ventilator time. Part of the reason may be that the samples were taken on ICU admission or later, meaning the sample represented a select population of severely ill COVID-19 patients with a long expected length of stay. This selection bias would be more marked with every consecutive sample.

Patients with missing bio-ADM values did not differ in demographics or comorbidities, but they had markedly shorter IMV times and spent less time in the ICU and hospital. Many of these patients were deceased or discharged from the ICU before a day 7 sample could be taken, resulting in a shorter length of stay and duration of mechanical ventilation. Although the study aimed to collect blood samples from patients discharged before day 7, this was sometimes difficult to achieve during the height of the Covid pandemic.

This study is the most extensive study on bio-ADM and COVID-19 to date, and the results were comparable to those of similar studies on bio-ADM and adverse outcomes in severe COVID-19. Although the predictive value of bio-ADM on mortality was fair at best, these results and the strong association with the need for renal replacement therapy indicate that adrenomedullin has a role in COVID-19 pathophysiology and measuring adrenomedullin levels could aid in predicting who will develop severe disease and multi-organ failure. In particular, individual trends in bio-ADM values may be important. Bio-ADM also represents a possible target pathway for interventions. Adrezucimab is an available treatment that improves endothelial stability by increasing plasma bio-ADM, warranting further studies of the connection between bio-ADM and severe respiratory diseases such as COVID-19. It is not clear why bio-ADM was inferior to MR-proADM in predicting mortality. It may be due to differences in sampling times and study populations, making a direct comparison difficult.

## Conclusion

In this study, serial bio-ADM samples in the ICU had limited predictive power for mortality in critically ill COVID-19 patients when looking at absolute values. Still, relative changes over time showed a much better prediction. Bio-ADM did not predict severe ARDS or length of stay, which may be due to the limited sample size. There was an association between bio-ADM and AKI, and bio-ADM predicted the need for renal replacement therapy. Further studies are warranted to investigate the pathophysiological role of bio-ADM in COVID-19 and develop prediction models or aid the development of future treatments.

## Data Availability

The datasets generated and analyzed during the current study are not publicly available due to limitations in the ethical approval of the study and data management policies of Region Skåne. However, they are available from the corresponding author upon reasonable request.

## References

[1] Ranieri, V. M. et al. Acute respiratory distress syndrome: The Berlin definition. *JAMA* **307**, 2526–2533 (2012). 22797452 10.1001/jama.2012.5669

[2] Didriksson, I. et al. Intensive care unit burden is associated with increased mortality in critically ill COVID-19 patients. *Acta Anaesthesiol. Scand.* **67**, 329–338 (2023). 36537243 10.1111/aas.14184 PMC9878196

[3] *NIH. Clinical Spectrum of SARS-CoV-2 Infection* (2023).

[4] Hoffmann, M. et al. SARS-CoV-2 cell entry depends on ACE2 and TMPRSS2 and is blocked by a clinically proven protease inhibitor. *Cell* **181**, 271–280 (2020). 32142651 10.1016/j.cell.2020.02.052 PMC7102627

[5] Ferrario, C. M. et al. Effect of angiotensin-converting enzyme inhibition and angiotensin II receptor blockers on cardiac angiotensin-converting enzyme 2. *Circulation* **111**, 2605–2610 (2005). 15897343 10.1161/CIRCULATIONAHA.104.510461

[6] Varga, Z. et al. Endothelial cell infection and endotheliitis in COVID-19. *Lancet* **395**, 1417–1418 (2020). 32325026 10.1016/S0140-6736(20)30937-5 PMC7172722

[7] Dirican, A., Ildir, S., Uzar, T., Karaman, I. & Ozkaya, S. 190 patients and literature review for a pathophysiological map to clinical categorisation. *Int. J. Clin. Pract.* **75**, e14843 (2021). 34519155 10.1111/ijcp.14843 PMC8646438

[8] Flaumenhaft, R., Enjyoji, K. & Schmaier, A. A. Vasculopathy in COVID-19. *Blood* **140**, 222–235 (2022). 34986238 10.1182/blood.2021012250 PMC8736280

[9] Hippenstiel, S. et al. Adrenomedullin reduces endothelial hyperpermeability. *Circ. Res.* **91**, 618–625 (2002). 12364390 10.1161/01.res.0000036603.61868.f9

[10] Kitamura, K. et al. Adrenomedullin: A novel hypotensive peptide isolated from human pheochromocytoma. 1993. *Biochem. Biophys. Res. Commun.* **425**, 548–555 (2012). 22925672 10.1016/j.bbrc.2012.08.022

[11] Jougasaki, M. & Burnett, J. C. Adrenomedullin: Potential in physiology and pathophysiology. *Life Sci.* **66**, 855–872 (2000). 10714887 10.1016/s0024-3205(99)00358-6

[12] Martinez, A., Miller, M. J., Unsworth, E. J., Siegfried, J. M. & Cuttitta, F. Expression of adrenomedullin in normal human lung and in pulmonary tumors. *Endocrinology* **136**, 4099–4105 (1995). 7649118 10.1210/endo.136.9.7649118

[13] Voors, A. A. et al. Adrenomedullin in heart failure: Pathophysiology and therapeutic application. *Eur. J. Heart Fail.* **21**, 163–171 (2019). 30592365 10.1002/ejhf.1366 PMC6607488

[14] Cockcroft, J. R., Noon, J. P., Gardner-Medwin, J. & Bennett, T. Haemodynamic effects of adrenomedullin in human resistance and capacitance vessels. *Br. J. Clin. Pharmacol.* **44**, 57–60 (1997). 9241097 10.1046/j.1365-2125.1997.00622.x PMC2042810

[15] García Ponce, A. et al. Loss of cortactin causes endothelial barrier dysfunction via disturbed adrenomedullin secretion and actomyosin contractility. *Sci. Rep.* **6**, 29003 (2016). 27357373 10.1038/srep29003 PMC4928053

[16] Weber, J. et al. Sandwich immunoassay for bioactive plasma adrenomedullin. *J. Appl. Lab. Med.* **2**, 222–233. 10.1373/jalm.2017.023655 (2019). https://academic.oup.com/jalm/article-pdf/2/2/222/31433623/jalm0222.pdf. 10.1373/jalm.2017.023655 32630976

[17] Marino, R. et al. Plasma adrenomedullin is associated with short-term mortality and vasopressor requirement in patients admitted with sepsis. *Crit. Care* **18**, R34 (2014). 24533868 10.1186/cc13731 PMC4056312

[18] Mebazaa, A. et al. Circulating adrenomedullin estimates survival and reversibility of organ failure in sepsis: The prospective observational multinational Adrenomedullin and Outcome in Sepsis and Septic Shock-1 (AdrenOSS-1) study. *Crit. Care* **22**, 354 (2018). 30583748 10.1186/s13054-018-2243-2 PMC6305573

[19] Lundberg, O. H. M. et al. Circulating bioactive adrenomedullin as a marker of sepsis, septic shock and critical illness. *Crit. Care* **24**, 636 (2020). 33148300 10.1186/s13054-020-03351-1 PMC7641835

[20] Geven, C., Bergmann, A., Kox, M. & Pickkers, P. Vascular effects of adrenomedullin and the anti-adrenomedullin antibody Adrecizumab in Sepsis. *Shock* **50**, 132–140 (2018). 29324626 10.1097/SHK.0000000000001103

[21] de Guadiana-Romualdo, L. et al. MR-proADM as marker of endotheliitis predicts COVID-19 severity. *Eur. J. Clin. Investig.* **51**, e13511 (2021). 33569769 10.1111/eci.13511 PMC7995076

[22] Gregoriano, C. et al. The vasoactive peptide MR-pro-adrenomedullin in COVID-19 patients: An observational study. *Clin. Chem. Lab. Med.* **59**, 995–1004 (2021). 33554516 10.1515/cclm-2020-1295

[23] Moore, N. et al. Mid-regional proadrenomedullin (MR-proADM), C-reactive protein (CRP) and other biomarkers in the early identification of disease progression in patients with COVID-19 in the acute NHS setting. *J. Clin. Pathol.* **76**, 400–406 (2023). 34996755 10.1136/jclinpath-2021-207750

[24] Sozio, E. et al. Identification of COVID-19 patients at risk of hospital admission and mortality: A European multicentre retrospective analysis of mid-regional pro-adrenomedullin. *Respir. Res.* **23**, 221 (2022). 36031619 10.1186/s12931-022-02151-1 PMC9420187

[25] van Lier, D. et al. Circulating dipeptidyl peptidase 3 and bio-adrenomedullin levels are associated with impaired outcomes in critically ill COVID-19 patients: A prospective international multicentre study. *ERJ Open Res.* **9**, 00342–02022 (2023). 36628268 10.1183/23120541.00342-2022 PMC9571166

[26] Simon, T. P. et al. Prognostic value of bioactive adrenomedullin in critically Ill patients with COVID-19 in Germany: An observational cohort study. *J. Clin. Med.* **10**, 1667 (2021). 33924637 10.3390/jcm10081667 PMC8069401

[27] Karakas, M. et al. Targeting endothelial dysfunction in eight extreme-critically Ill patients with COVID-19 using the anti-adrenomedullin antibody Adrecizumab (HAM8101). *Biomolecules* **10**, 1171 (2020). 32796765 10.3390/biom10081171 PMC7465983

[28] Fialek, B. et al. Systematic review with meta-analysis of mid-regional pro-adrenomedullin (MR-proADM) as a prognostic marker in COVID-19-hospitalized patients. *Ann. Med.* **55**, 379–387 (2023). 36607317 10.1080/07853890.2022.2162116 PMC9828692

[29] Sievert, T. et al. Neurofilament light chain on intensive care admission is an independent predictor of mortality in COVID-19: A prospective multicenter study. *Intensive Care Med. Exp.* **11**, 66 (2023). 37768470 10.1186/s40635-023-00547-x PMC10539241

[30] von Elm, E. et al. The strengthening the reporting of observational studies in epidemiology (STROBE) statement: Guidelines for reporting observational studies. *Lancet* **370**, 1453–1457 (2007). 18064739 10.1016/S0140-6736(07)61602-X

[31] Charlson, M. E., Pompei, P., Ales, K. L. & MacKenzie, C. R. A new method of classifying prognostic comorbidity in longitudinal studies: Development and validation. *J. Chronic Dis.* **40**, 373–383 (1987). 3558716 10.1016/0021-9681(87)90171-8

[32] Moreno, R. P. et al. SAPS 3–From evaluation of the patient to evaluation of the intensive care unit. Part 2: Development of a prognostic model for hospital mortality at ICU admission. *Intensive Care Med.* **31**, 1345–1355 (2005). 16132892 10.1007/s00134-005-2763-5 PMC1315315

[33] Vincent, J. L. et al. The SOFA (Sepsis-related Organ Failure Assessment) score to describe organ dysfunction/failure. On behalf of the Working Group on Sepsis-Related Problems of the European Society of Intensive Care Medicine.. *Intensive Care Med.* **22**, 707–710 (1996). 8844239 10.1007/BF01709751

[34] Kellum, J. A., Lameire, N. & Group, K. A. G. W. Diagnosis, evaluation, and management of acute kidney injury: A KDIGO summary (Part 1). *Crit. Care* **17**, 1–15 (2013). 10.1186/cc11454 PMC4057151 23394211

[35] Grasselli, G. et al. ESICM guidelines on acute respiratory distress syndrome: Definition, phenotyping and respiratory support strategies. *Intensive Care Med.* **49**, 727–759 (2023). 37326646 10.1007/s00134-023-07050-7 PMC10354163

[36] Xie, Z. et al. Adrenomedullin surges are linked to acute episodes of the systemic capillary leak syndrome (Clarkson disease). *J. Leukoc. Biol.* **103**, 749–759 (2018). 29360169 10.1002/JLB.5A0817-324R PMC6312640

[37] Vieceli Dalla Sega, F. et al. ime course of endothelial dysfunction markers and mortality in COVID-19 patients: A pilot study. *Clin. Transl. Med.* **11**, e283 (2021). 33784001 10.1002/ctm2.283 PMC7919132

[38] Kozhuharov, N. et al. Activity of the adrenomedullin system to personalise post-discharge diuretic treatment in acute heart failure. *Clin. Res. Cardiol.* **111**, 627–637 (2022). 34302189 10.1007/s00392-021-01909-9 PMC9151518

[39] Roedl, K. et al. MR-proAdrenomedullin as a predictor of renal replacement therapy in a cohort of critically ill patients with COVID-19. *Biomarkers* **26**, 417–424 (2021). 33754916 10.1080/1354750X.2021.1905067

[40] Nadim, M. K. et al. COVID-19-associated acute kidney injury: Consensus report of the 25th Acute Disease Quality Initiative (ADQI) Workgroup. *Nat. Rev. Nephrol.* **16**, 747–764 (2020). 33060844 10.1038/s41581-020-00356-5 PMC7561246

