# Author Correction: Plasma bioactive adrenomedullin predicts mortality and need for dialysis in critical COVID-19

**DOI:** 10.1038/s41598-025-00667-2

**Published:** 2025-05-12

**Authors:** Patrik Johnsson, Theodor Sievert, Ingrid Didriksson, Hans Friberg, Attila Frigyesi

**Affiliations:** 1 Department of Clinical Medicine, Anaesthesiology and Intensive Care, Lund University, 22185 Lund, Sweden; 2 Department of Intensive and Perioperative Care in Malmö, Skåne University Hospital, 20502 Malmö, Sweden; 3 Department of Intensive and Perioperative Care in Lund, Skåne University Hospital, 22185 Lund, Sweden

Correction to: *Scientific Reports* 10.1038/s41598-024-74380-x, published online 11 October 2024

The original version of this Article contained an error in Figure 3 and 4 where the graphs were incorrectly labelled. The original Figure 3 and 4 and the accompanying legends appear below.

**Fig. 3:**
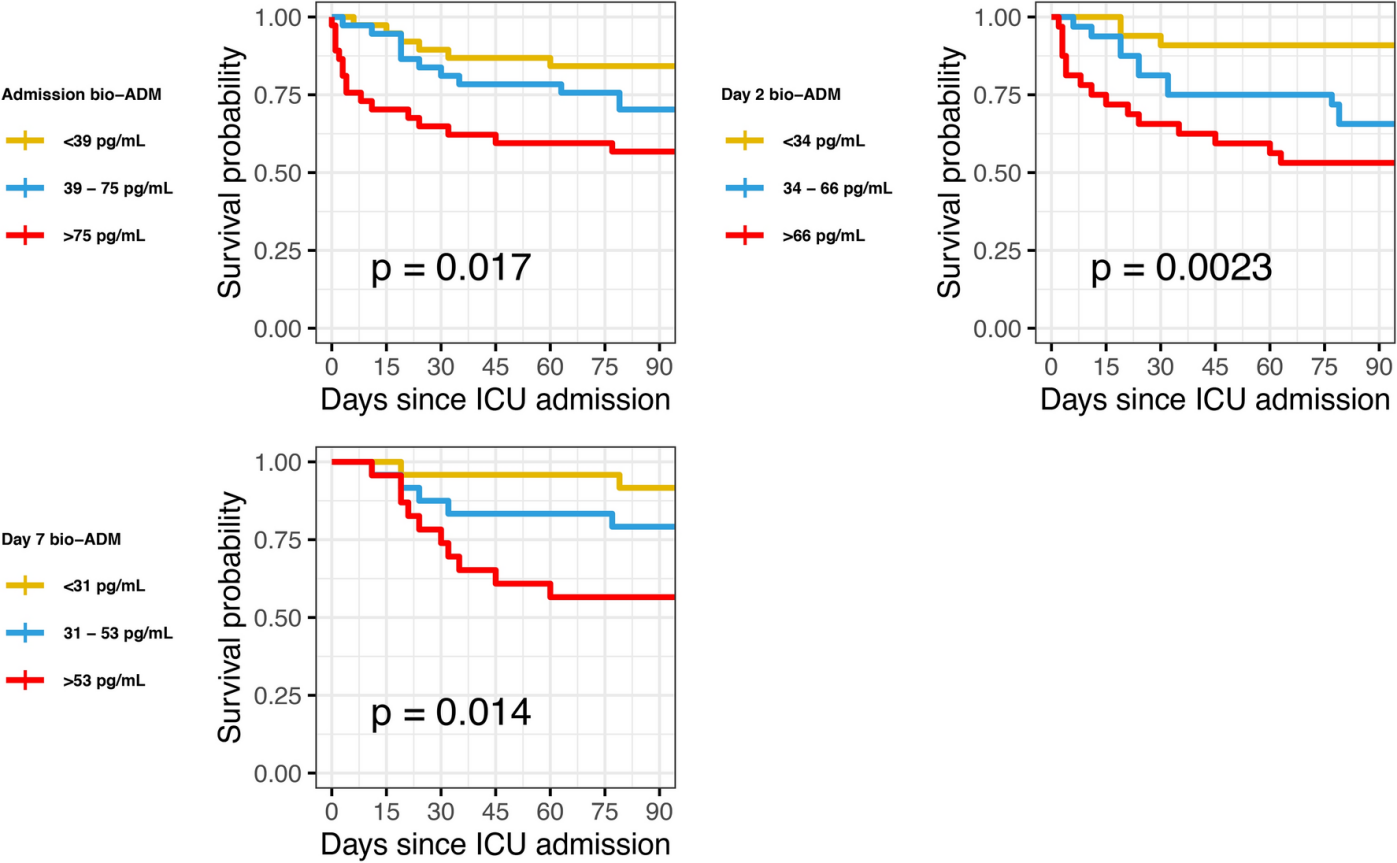
Bio-ADM tertiles and survival in severe COVID-19, from ICU admission, day 2, and day 7. Bio-ADM: circulating bioactive adrenomedullin; ICU: intensive care unit.

**Fig. 4:**
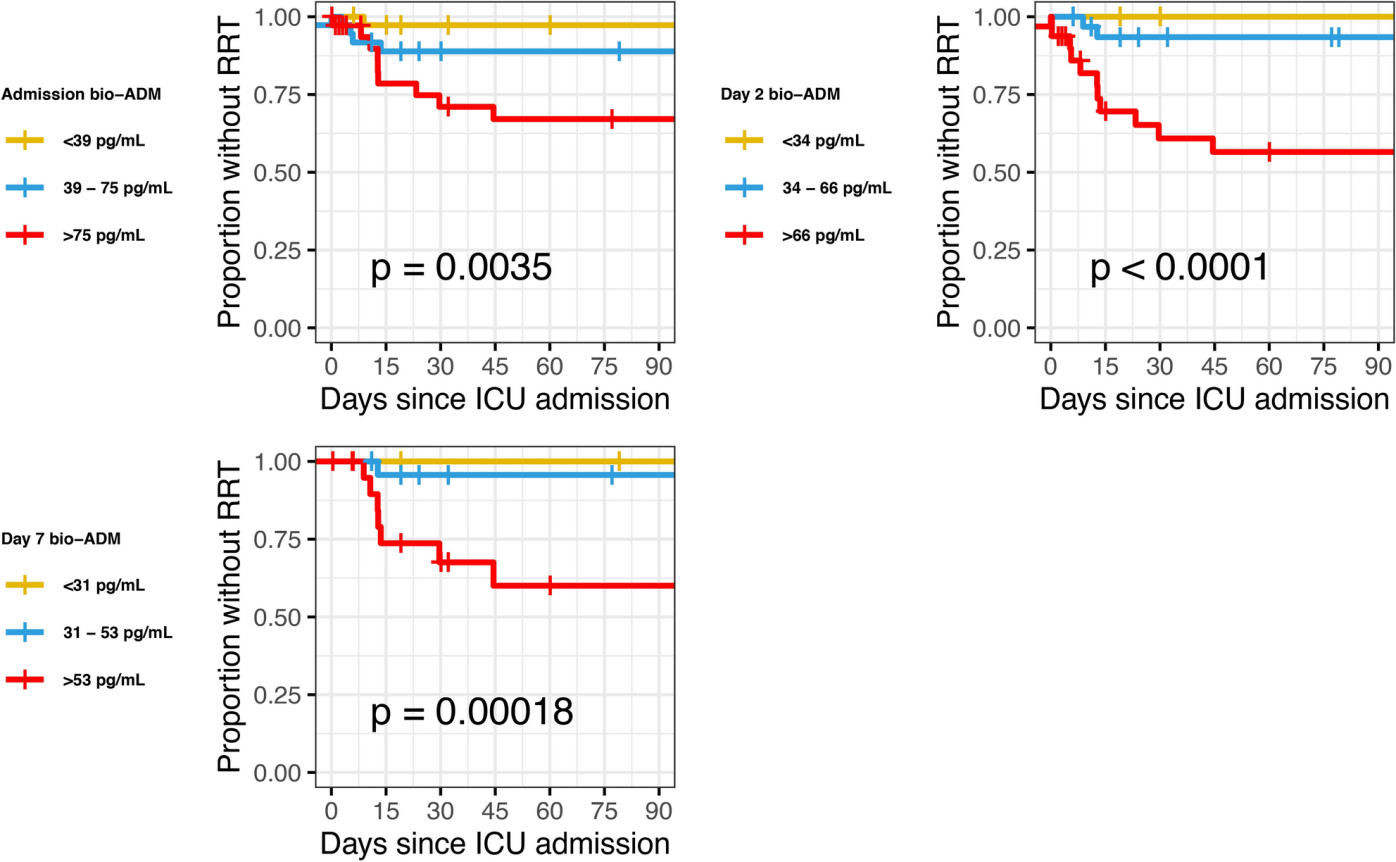
Bio-ADM tertiles and RRT-free survival, from ICU admission, day 2, and day 7. Bio-ADM: circulating bioactive adrenomedullin; RRT: renal replacement therapy; ICU: intensive care unit.

The original Article has been corrected.

